# Obatoclax impairs lysosomal function to block autophagy in cisplatin-sensitive and -resistant esophageal cancer cells

**DOI:** 10.18632/oncotarget.7492

**Published:** 2016-02-19

**Authors:** Le Yu, William KK Wu, Chunping Gu, Desheng Zhong, Xuyan Zhao, Yi Kong, Qinghuan Lin, Matthew TV Chan, Zhitao Zhou, Shuwen Liu

**Affiliations:** ^1^ State Key Laboratory of Organ Failure Research, Guangdong Provincial Key Laboratory of New Drug Screening, School of Pharmaceutical Sciences, Southern Medical University, Guangzhou, China; ^2^ Department of Anaesthesia and Intensive Care, The Chinese University of Hong Kong, Hong Kong, China; ^3^ State Key Laboratory of Digestive Diseases, LKS Institute of Health Sciences, The Chinese University of Hong Kong, Hong Kong, China; ^4^ Nanfang Hospital, Southern Medical University, Guangzhou, China; ^5^ Electron Microscopy laboratory, Southern Medical University, Guangzhou, China

**Keywords:** autophagy, lysosome, obatoclax, cisplatin resistance, cathepsin

## Abstract

Obatoclax, a pan-inhibitor of anti-apoptotic Bcl-2 proteins, exhibits cytotoxic effect on cancer cells through both apoptosis-dependent and -independent pathways. Here we show that obatoclax caused cytotoxicity in both cisplatin-sensitive and -resistant esophageal cancer cells. Although obatoclax showed differential apoptogenic effects in these cells, it consistently blocked autophagic flux, which was evidenced by concomitant accumulation of LC3-II and p62. Obatoclax was trapped in lysosomes and induced lysosome clustering. Obatoclax also substantially reduced the expression of lysosomal cathepsins B, D and L. Moreover, cathepsin knockdown was sufficient to induce cytotoxicity, connecting lysosomal function to cell viability. Consistent with the known function of autophagy, obatoclax caused the accumulation of polyubiquitinated proteins and showed synergy with proteasome inhibition. Taken together, our studies unveiled impaired lysosomal function as a novel mechanism whereby obatoclax mediates its cytotoxic effect in esophageal cancer cells.

## INTRODUCTION

Obatoclax mesylate is an intravenously-administered drug under investigation in Phase I and II clinical trials as a novel anticancer agent. Obatoclax is proposed to act as a pan-inhibitor of anti-apoptotic members of the Bcl- 2 family, which antagonize the mitochondrial pathway of apoptosis [1]. The Bcl-2 family consists of anti-apoptotic proteins (e.g., Bcl-2, Mcl-1, Bcl-xL) and two subfamilies of pro-apoptotic proteins, namely the pore-forming Bak and Bax and the BH3-only proteins (e.g. Bad, Bim, Bid, Puma, Noxa). BH3-only proteins sense apoptotic stimuli and act as activators and/or sensitizers in the process of Bax- and Bak-mediated permeabilization of the outer mitochondrial membrane, while the anti-apoptotic Bcl-2 family inhibits this process [2, 3]. Overexpression of Bcl-2 and related anti-apoptotic family members may lead to an increase in the apoptotic threshold of cancer cells and has been linked to chemoresistance and poor clinical outcomes [4–6]. Thus, inhibitors of anti-apoptotic members of the Bcl-2 family, such as obatoclax, represent attractive agents for subverting related drug resistance.

Cisplatin is a widely used chemotherapeutic agent [7]. Unfortunately, the development of cisplatin resistance in cancer cells represents a major barrier for successful treatment outcomes [8]. Identification of new drugs that could overcome cisplatin resistance is an area of active investigation. In this scenaria, obatoclax in combination with cisplatin or cisplatin-containing regimen has been shown to synergistically reduce the viability of oral and lung cancer cells [9–11]. Moreover, cisplatin-resistant non-small cell lung cancer cells not only showed sensitivity to obatoclax *in vitro* [12], but also responded obatoclax as monotherapy in xenotransplanted mice [13]. Nevertheless, the molecular mechanism underlying obatoclax in cisplatin-resistant cancer cells remains obscure.

Many studies have shown that obatoclax induces apoptosis by suppressing anti-apoptotic family members of Bcl-2 [14–17]. However, the cytotoxicity of obatoclax has also been observed in Bax- and Bak-deficient cells, suggesting the existence of mechanism independent of the mitochondrial pathway of apoptosis [16, 18, 19]. In this respect, emerging evidence hints at the involvement of autophagy in the cytotoxic action of obatoclax [20]. However, the direct influence of obatoclax on autophagy remains controversial, since both autophagy-promoting and -suppressing effects have been reported [21–28]. Here we show that obatoclax as a single agent could induce equivalent loss of cell viability in cisplatin-sensitive and -resistant esophageal cancer cells. Interestingly, obatoclax impairs lysosomal functions in these cells, leading to the blockage of autophagic flux.

## RESULTS

### Obatoclax reduced cell viability equally in cisplatin-sensitive and -resistant esophageal cancer cells

**Figure 1:**
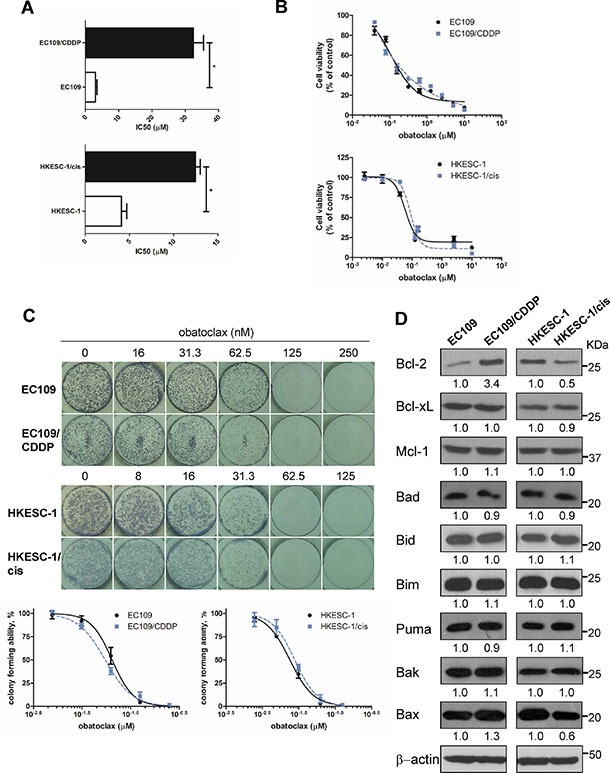
Obatoclax reduced cell viability of both cisplatin-sensitive and –resistant esophageal cancer cells (**A**) Parental and cisplatin-resistant esophageal cancer cells (EC109 and its resistant subline EC109/CDDP, HKESC-1 and its resistant subline HKESC-1/cis) were treated with cisplatin (0–160 μM) for 48 h. Cell viability was then determined by MTT assay. IC_50_ values were calculated with Prism software. Data are presented as the mean ± S.E.M. from three independent experiments. *P < 0.05 compared with the respective parental cell line. (**B**) Cells were exposed to increasing concentrations of obatoclax for 48 h. Cell viability was then determined by MTT assay. Data are presented as the mean ± S.E.M. (*n* = 3) of a representative experiment performed in triplicate. (**C**) Cells were treated with obatoclax at the indicated concentrations for 48 h. Viable, adherent cells were counted and re-seeded (3,000 cells per well) into a well of a six-well plate (in triplicate), in the absence of obatoclax. Ten to twelve days later, colonies were fixed and stained. Each well shown is a representative image of at least nine similar wells (three independent experiments). Data are presented as the mean ± S.E.M. (*n* = 3) of a representative experiment performed in triplicate. (**D**) The basal expression levels of Bcl-2 family members were measured by Western blots. β-actin was used to evaluate protein loading. Blots were representative of 3 independent experiments. Quantification of the ratios of Bcl-2/β-actin, Bcl-xL/β-actin, Mcl-1/β-actin, Bad/β-actin, Bid/β-actin, Bim/β-actin, Puma/β-actin, Bak/β-actin, and Bax/β-actin is shown below each gel lane. The ratios were normalized to parental cell lines EC109 and HKESC-1 cells, respectively.

To determine whether obatoclax could exhibit cytotoxic action in esophageal cancer cells with cisplatin resistance, two pairs of parental and cisplatin-resistant esophageal cancer cell lines (EC109 and its resistant subline EC109/CDDP; HKESC-1 and its resistant subline HKESC-1/cis) were utilized in our study. At the time of investigation, EC109/CDDP was about 11-fold resistant to cisplatin than the parental cell line EC109, as evidenced by an IC_50_ (48 h) of 32.4 ± 3.1 μM versus 3.0 ± 0.1 μM, respectively. The IC_50_ (48 h) for HKESC-1/cis and its parental cell line HKESC-1 was 12.5 ± 0.1 μM and 4.1 ± 0.1 μM respectively, showing 3-fold difference in cisplatin sensitivity (Figure 1A). The IC_50_ (48 h) for obatoclax was also determined in these cell lines. The IC_50_ values of obatoclax were 0.24 ± 0.04 μM and 0.29 ± 0.01 μM for EC109 and EC109/CDDP cells, respectively. Likewise, obatoclax reduced cell viability of HKESC-1 and HKESC-1/cis cells to a similar extent with the same IC_50_ value of 0.13 ± 0.02 μM for both cell lines (Figure 1B). To investigate the long-term effect of obatoclax, colony formation assay was performed. The IC_50_ values for EC109 and EC109/CDDP were 0.064 ± 0.006 μM and 0.056 ± 0.004 μM, respectively. Also, obatoclax similarly decreased colony-forming ability of HKESC-1 and HKESC-1/cis cells with IC_50_ values of 0.024 ± 0.001 μM and 0.027 ± 0.002 μM, respectively (Figure 1C). These results strongly suggest that obatoclax as a single agent is capable of inducing the loss of cell viability and decreasing self-renewal capacity in both cisplatin-sensitive and -resistant esophageal cancer cells.

### Bcl-2 family expression in cisplatin-sensitive and –resistant esophageal cancer cell lines

Expression patterns of key Bcl-2 family members, including anti-apoptotic proteins (Bcl-2, Bcl-xL, and Mcl- 1) and pro-apoptotic proteins (Bad, Bid, Bim, Puma, Bak, and Bax), were compared between cisplatin-resistant cell lines and their sensitive counterparts. Bcl-2 expression was elevated in EC109/CDDP cells compared to EC109 cells, whereas it was decreased in HKESC-1/cis cells compared with HKESC-1 cells. There was no obvious change in Bcl-xL and Mcl-1 expression between cisplatin-resistant cell lines and their parental cell lines. When it comes to pro-apoptotic proteins, the expression of Bad, Bid, Bim, Puma, Bak in cisplatin-resistant cell lines was similar to their parental cell lines. Nonetheless, Bax level was increased in EC109/CDDP cells compared to EC109 cells, while it was decreased in HKESC-1/cis cells compared with HKESC-1 cells (Figure 1D). These data show that the changes in protein expression of Bcl-2 family members vary in different cisplatin-resistant cell lines compared to their sensitive counterparts.

### Obatoclax induced apoptosis in EC109, EC109/CDDP and HKESC-1 but not HKESC-1/cis

**Figure 2:**
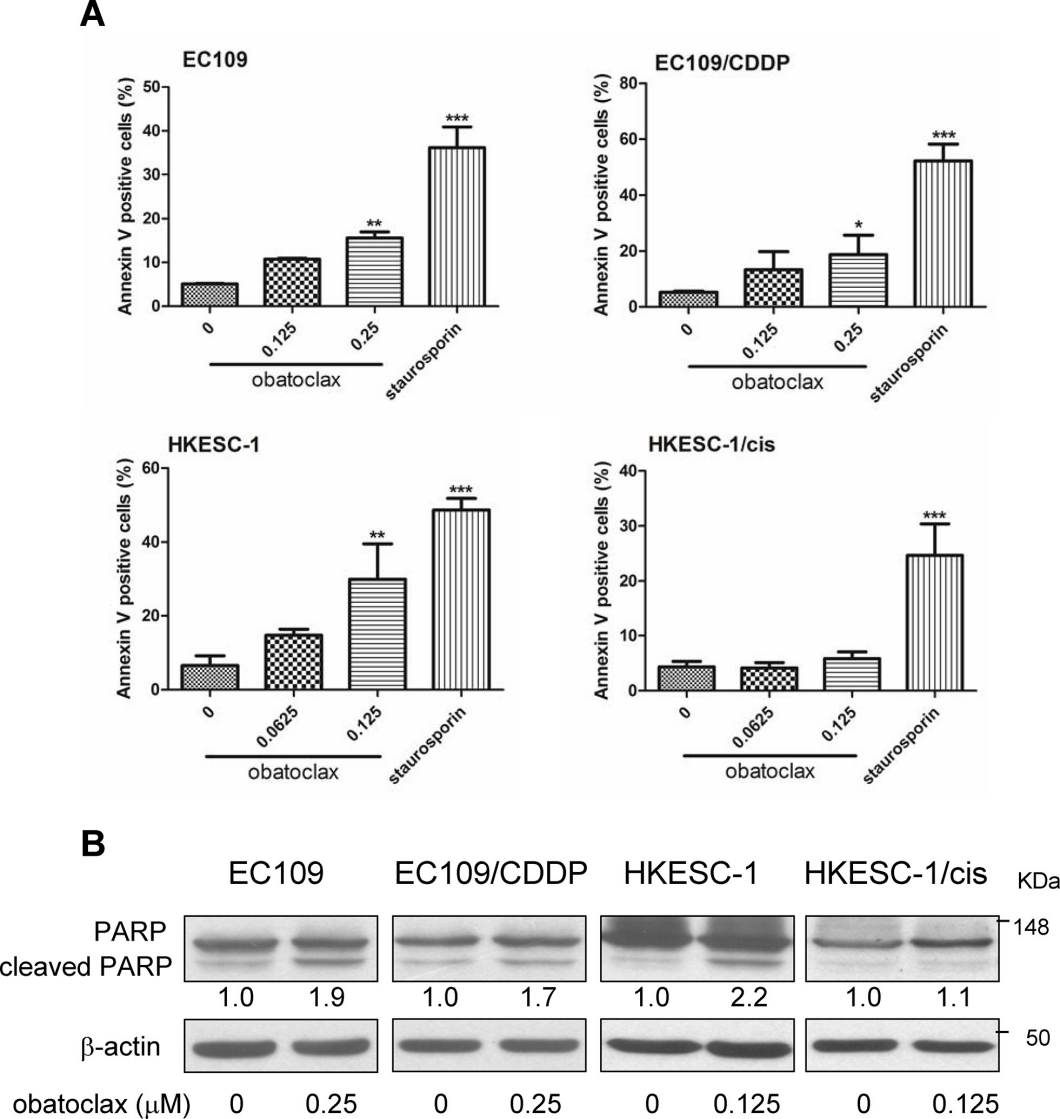
Effects of obatoclax on apoptosis (**A**) Parental and cisplatin-resistant esophageal cancer cells (EC109 and its resistant subline EC109/CDDP, HKESC-1 and its resistant subline HKESC-1/cis) were exposed to the indicated concentrations of obatoclax for 48 h. Cellular apoptosis was assessed by phosphatidylserine (PS) externalization and binding of Annexin V-FITC. Data are presented as mean ± S.E.M. from three independent experiments. *P < 0.05, **P < 0.01, ***P < 0.001 as compared to the control. Staurosporin (50 nM) was used as a positive control. (**B**) Cells were treated with the indicated concentrations of obatoclax for 48 h. PARP cleavage was examined by Western blots. β-actin was used to evaluate protein loading. Blots were representative of 3 independent experiments. Quantification of the ratios of cleaved PARP/β-actin is shown below each gel lane. The ratios were normalized to control cells.

To determine whether apoptosis contributes to the reduced cell viability induced by obatoclax, we performed Annexin V labeling in cells treated with or without obatoclax for 48 h. Obatoclax at a concentration of 0.25 μM (approximate its IC_50_ for EC109 and EC109/CDDP cells) significantly increased population of Annexin V-positive cells in EC109 and EC109/CDDP cells. In contrast, obatoclax at a concentration of 0.125 μM (approximate its IC_50_ for HKESC-1 and HKESC-1/cis cells) significantly increased apoptosis in HKESC-1 cells, whereas it failed to promote apoptosis in HKESC-1/cis cells. Staurosporin (50 nM), which was used as a positive control for inducing apoptosis, robustly increased population of Annexin V-positive cells in all tested cell lines (Figure 2A). We further confirmed the effect of obatoclax on apoptosis by determination of PARP cleavage, which is a molecular marker of apoptosis. Like the results of Annexin V labeling experiment, obatoclax at the indicated concentrations promoted PARP cleavage in EC109, EC109/CDDP and HKESC-1 cells, whereas it exerted no effect in HKESC-1/cis cells (Figure 2B).

### Obatoclax blocked autophagic flux

**Figure 3:**
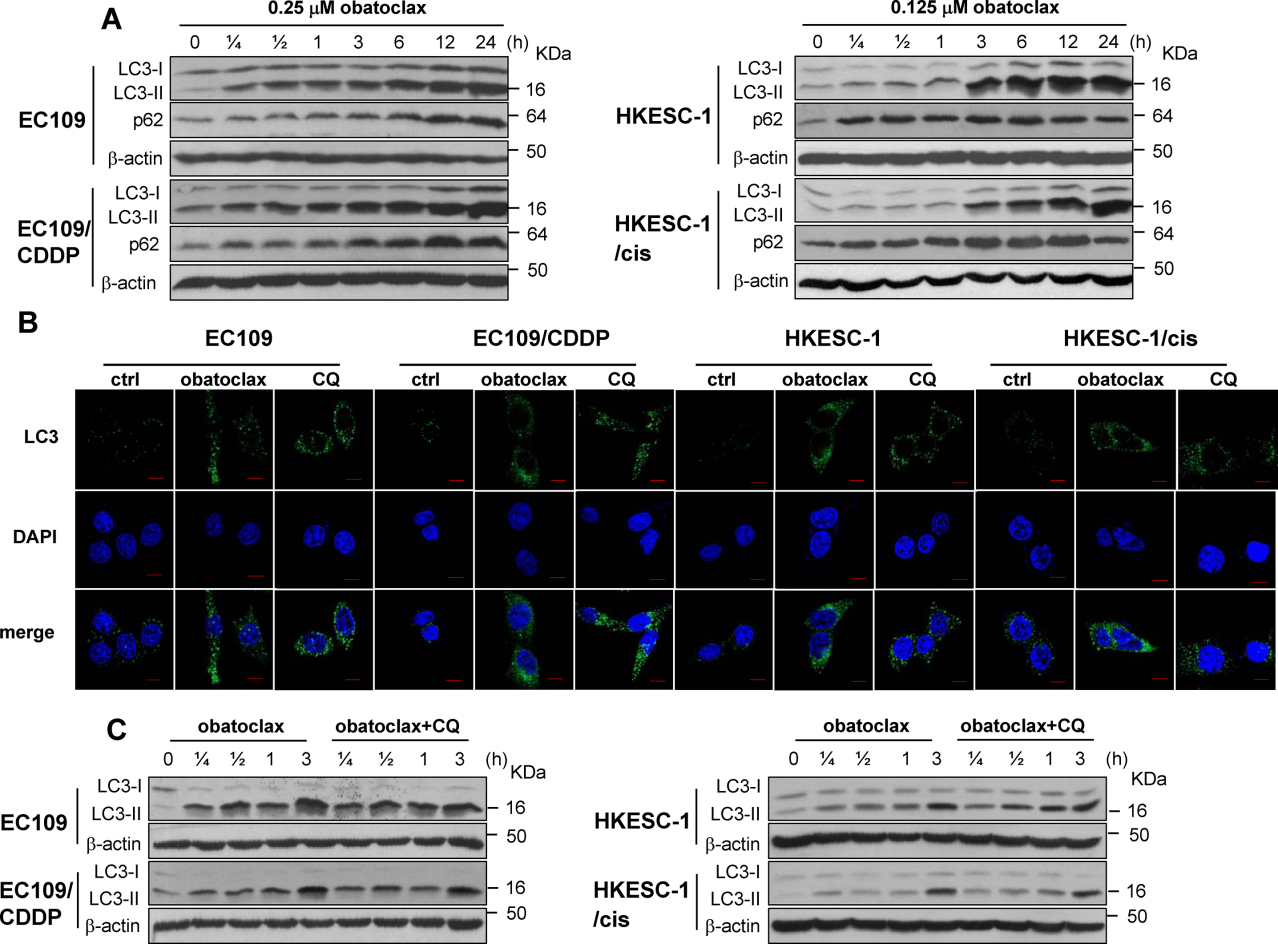
Obatoclax blocked autophagy flux (**A**) Cells were treated with obatoclax at the indicated concentrations for various time points. The conversion of LC3-I to LC3-II and the expression of p62 were determined by Western blot analysis. (**B**) Cells were treated with CQ (50 μM) or obatoclax at the concentration of 0.25 μM (EC109 and EC109/CDDP cells) and 0.125 μM (HKESC-1 and HKESC-1/cis cells) for 3 h. The formation of autophagic vacuoles was determined by immunofluorescent staining for LC3. Scale bars, 10 μm. (**C**) Cells were treated with obatoclax at the concentration of 0.25 μM (EC109 and EC109/CDDP cells) and 0.125 μM (HKESC-1 and HKESC-1/cis cells) in the absence of presence of CQ (50 μM) for the indicated time points. The conversion of LC3-I to LC3-II was determined by Western blot analysis. β-actin was used to evaluate protein loading. Blots were representative of 3 independent experiments.

**Figure 4:**
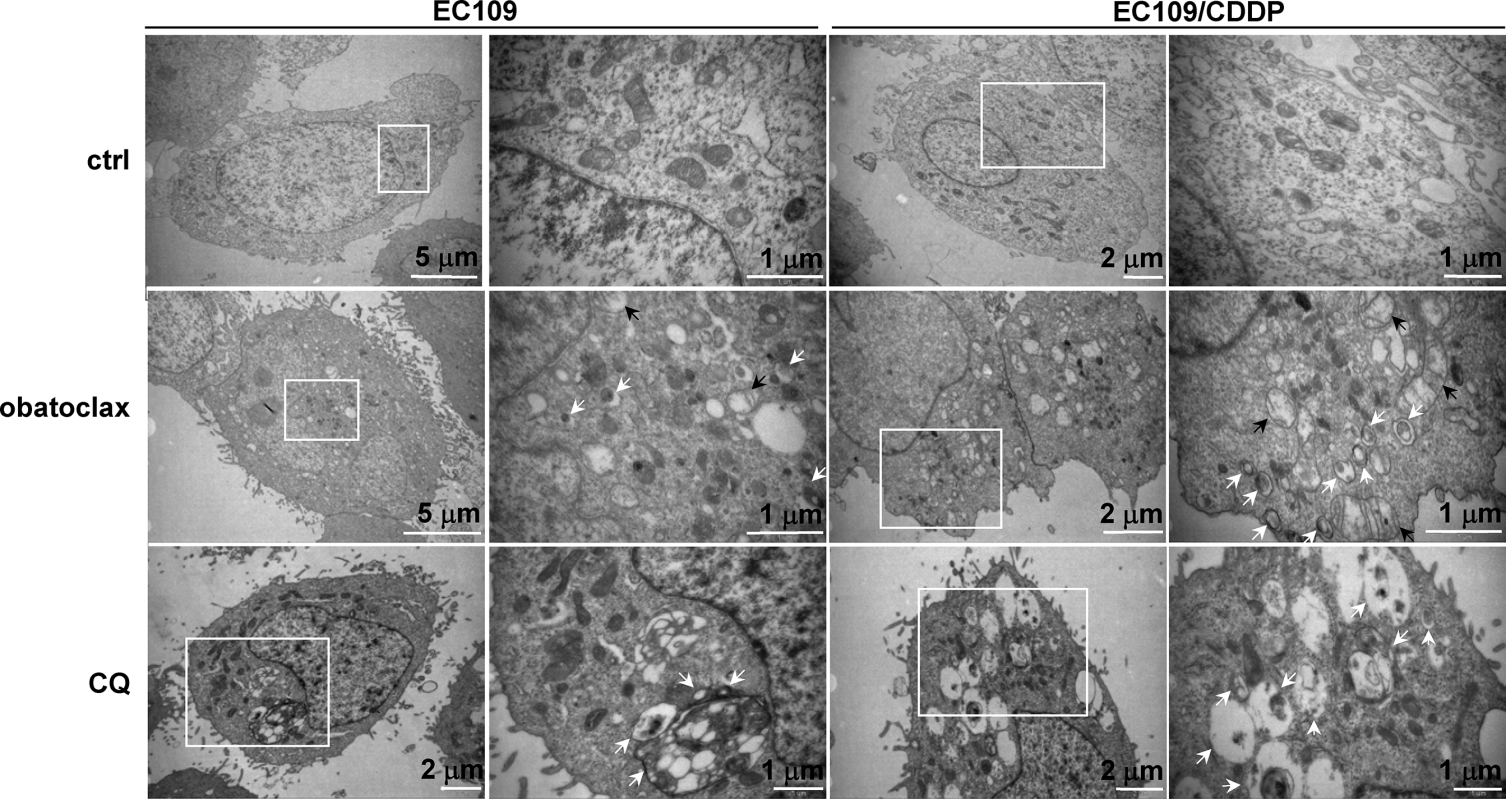
Electronic microscopy evidence of autophagy in obatolcax-treated cells EC109 and EC109/CDDP cells were treated with obatoclax (0.25 μM) or CQ (50 μM) for 12 hours and subjected to electron transmission microscopy. White arrows point to the autophagic vacuoles while black arrows point to the swollen mitochondria. For each treatment or control group, transmission electron microscope images were randomly chosen, from a field of at least 100 cells. Scale bars are included within the micrographs.

Autophagy has been implicated in modulating the cytotoxicity of obatoclax in various cancer cells [21–28]. We therefore studied the effect of obatoclax on autophagy by determining LC3-II and p62 protein expression at various time points within 24 h. LC3-II is an autophagosomal surface protein marker that is ultimately degraded inside the autolysosomes [29]. Western blots showed that the LC3-II level was dramatically increased by obatoclax treatment. Concordantly, the accumulation of p62, a protein degraded by autophagy [29], was observed in all tested cell lines. The massive increase in protein levels of LC3-II and p62 indicated that autophagic-lysosomal degradation activity was impaired by obatoclax treatment (Figure 3A). Immunofluorescence staining showed that obatoclax exposure for 3 h substantially induced the accumulation of LC3-positive autophagic vacuoles, phenocopying the effect of lysosome inhibitor chloroquine (CQ) in all tested cell lines (Figure 3B). In order to determine the effect of obatoclax on autophagic flux, CQ was used to inhibit lysosome and thus LC3- II degradation. The inhibitory effect of CQ on LC3 II degradation was confirmed by Western blots (Figure S1). Importantly, the addition of CQ did not alter obatoclax-induced LC3-II accumulation in EC109 and EC109/CDDP cells (Figure 3C). These findings suggest that obatoclax induced sustained LC3-II accumulation by blocking autophagic flux. In addition to biochemical analysis, we investigated the ultrastructure of cells treated with or without obatoclax for 12 h by transmission electron microscopy. Both EC109 and EC109/CDDP cells displayed extensive cytoplasmic vacuolization, with numerous vesicles engulfing cytoplasmic materials and components, which are typical structures of autophagosomes and autolysosomes. Similar vesicles were also evident in CQ-treated cells. In contrast, obatoclax induced mitochondrial swelling and loss of cristae, whereas CQ did not affect the ultrastructure of mitochondria. White and black arrows point to the autophagic vacuoles and the swollen mitochondria, respectively (Figure 4).

### Inhibition of basal autophagic activity blunted obatoclax-induced accumulation of LC3-II

**Figure 5:**
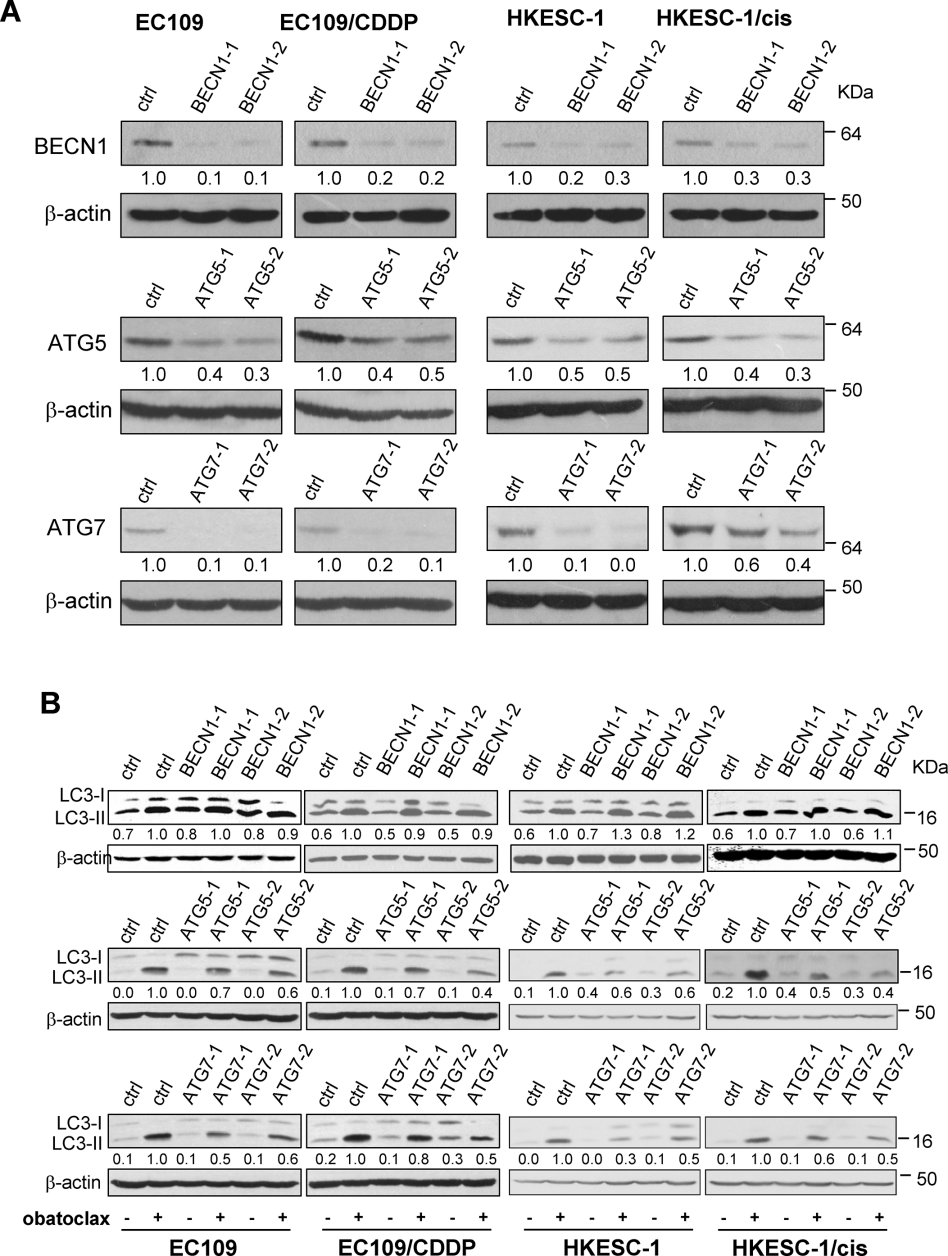
Effects of siRNA-mediated knockdown of Beclin-1, ATG5, or ATG7 on obatoclax-induced LC3-II accumulation (**A**) The efficacy of Beclin-1 (BECN1), ATG5, or ATG7 knockdown by respective siRNA was verified by Western blot analysis. Nontargeting siRNA was used as control siRNA, which has no homology to any known mammalian genes. Quantification of the ratios of Beclin-1/β-actin, ATG5/β-actin, and ATG7/β-actin is shown below each gel lane. The ratios were normalized to control siRNA-transfected cells. (**B**) After transfection with the control siRNA, Beclin-1 siRNA, ATG5 siRNA, or ATG7 siRNA, cells were treated with obatoclax at the concentration of 0.25 μM (EC109 and EC109/CDDP cells) and 0.125 μM (HKESC-1 and HKESC-1/cis cells) for 3 h. The conversion of LC3-I to LC3-II was determined by Western blot analysis. β-actin was used to evaluate protein loading. Blots were representative of 3 independent experiments. Quantification of the ratios of LC3-II/β-actin is shown below each gel lane. The ratios were normalized to control siRNA-transfected cells with obatoclax treatment.

We next investigated whether inhibition of basal autophagic activity could affect obatoclax-induced LC3- II accumulation. Beclin-1 is a key regulator of classical autophagy which is required at vesicle nucleation step. By contrast, ATG5 and ATG7 are known to function in autophagosome formation and completion [30]. Knockdown of these key regulators by siRNA was used to suppress basal autophagic activity by preventing autophagosome formation. As shown in Figure 5A, the silencing efficacy of Beclin-1, ATG5- and ATG7-siRNA was confirmed by Western blots. Downregulation of ATG5 or ATG7 reduced LC3-II accumulation induced by obatoclax treatment for 3 h, whereas knockdown of Beclin-1 failed to exert a similar effect (Figure 5B). Indeed, similar blunting effects of ATG5 and ATG7 knockdown were observed in CQ-treated cells (Figure S2). Also, knockdown of ATG5 or ATG7 decreased basal LC3- II expression (Figure S3). These findings suggest that basal autophagic activity was ATG5- and ATG7-dependent but not reliant on Beclin-1. Given that obatoclax phenocopied the effect of CQ in LC3-II accumulation when initiation of autophagy was inhibited, it is reasonable to speculate that obatoclax may block autophagy flux at the late degradation stage similar to CQ.

### Obatoclax was trapped in lysosomes

**Figure 6:**
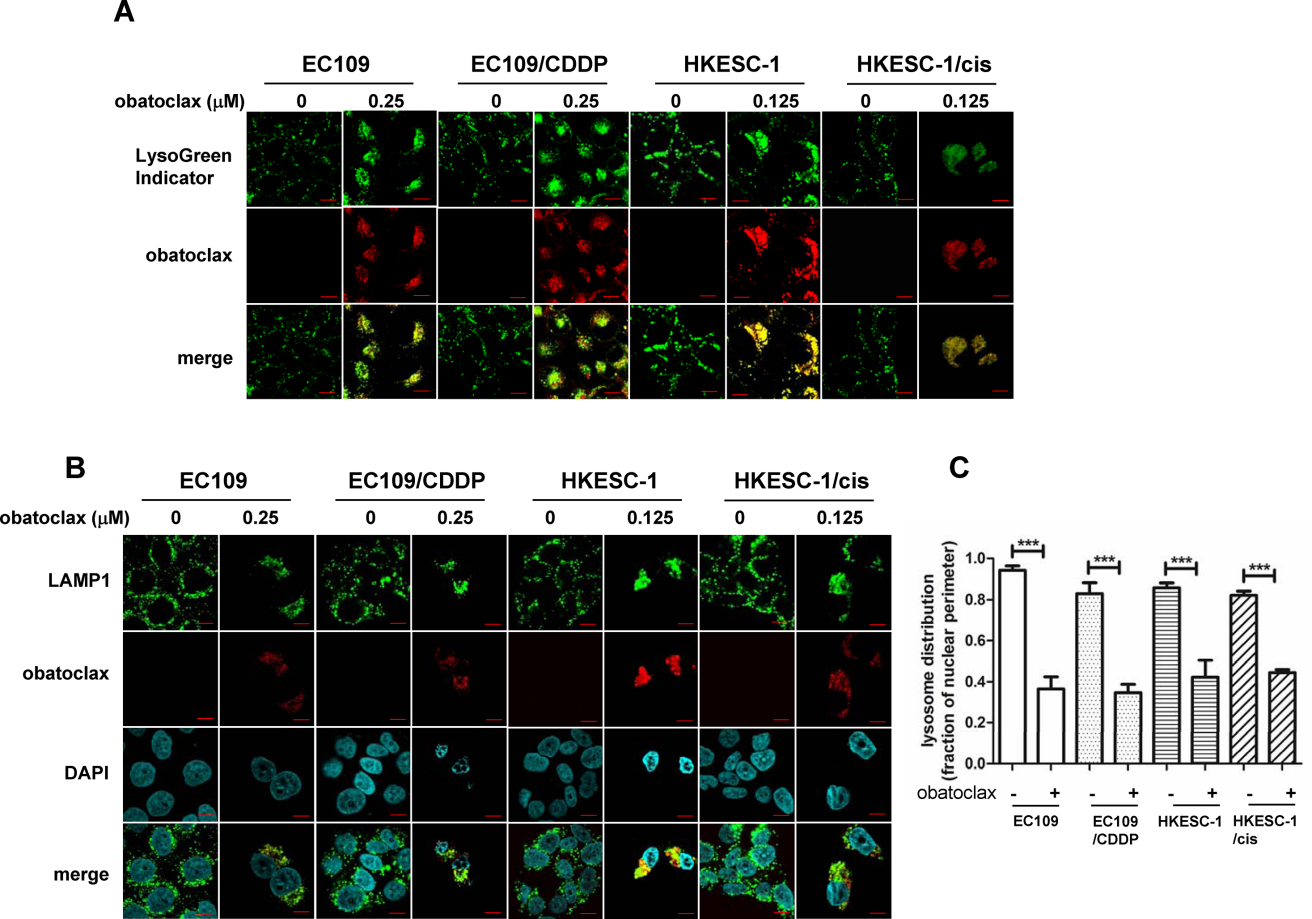
Intracellular distribution of obatoclax (**A**) Cells were treated with obatoclax at the indicated concentrations for 3 h. Lysosomes in live cells were stained with LysoGreen Indicator reagent. Scale bars, 10 μm. (**B**) Cells were treated with obatoclax at the indicated concentrations for 3 h. Immunofluorescent staining for LAMP1 marked lysosomes. The results shown are representative images. Scale bars, 10 μm. (**C**) Quantification of lysosome distribution (lysosome distribution relative to nuclear perimeter) is shown. For each point, at least 30 cells were pooled from three independent experiments. ***P < 0.001 as compared to control cells.

We next investigated whether obatoclax is a lysosomotropic agent like CQ. Obatoclax is an autofluorescent compound that emits red fluorescence upon excitation. This feature enables the visualization of its presence by confocal microscopy. CytoPainter LysoGreen Indicator is a fluorogenic probe, which can be used to label lysosomes in live cells. As shown in Figure 6A, obatoclax localized in lysosomes with a vesicular staining pattern after 3 h treatment. Using lysosome-associated membrane protein 1 (LAMP1) to mark lysosomes, we further confirmed that obatoclax became trapped in lysosomes after 3 h exposure (Figure 6B). Remarkably, obatoclax caused a marked redistribution of lysosomes. In this respect, lysosomes in untreated cells were dispersed evenly in the perinuclear region whereas obatoclax caused clustering of lysosomes in the cytosol (Figure 6A and 6B). Similarly, CQ also induced lysosome clustering (Figure S4).

### Obatoclax blocked autophagic degradation through inhibition of cathepsin levels

**Figure 7:**
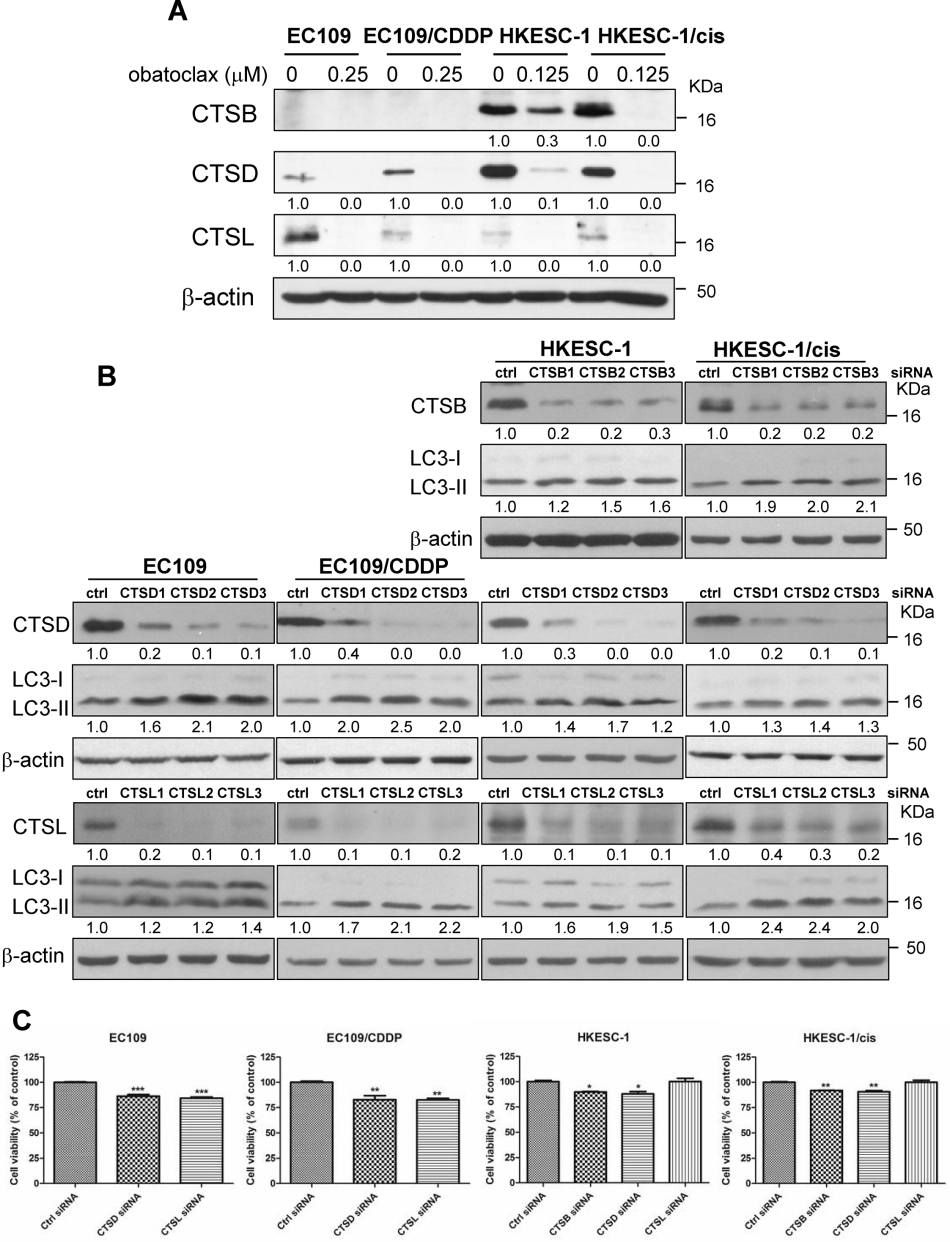
Effects of obatoclax on lysosomal functions (**A**) Cells were treated with obatoclax at the indicated concentrations for 48 h. The active forms of cathepsin B, D and L were determined by Western blot analysis. Quantification of the ratios of CTSB/β-actin, CTSD/β-actin, and CTSL/β-actin is shown below each gel lane. The ratios were normalized to control cells. (**B**) The efficacy of cathepsin B (CTSB), cathepsin D (CTSD), or cathepsin L (CTSL) by respective siRNA was confirmed by Western blot analysis at 48 h post-transfection. Nontargeting siRNA was used as control siRNA, which has no homology to any known mammalian genes. Targeting CTSB, D, or L by RNA interference increased the conversion of LC3-I to LC3-II at 48 h post-transfection. Quantification of the ratios of CTSB/β-actin, CTSD/β-actin, CTSL/β-actin and LC3-II/β-actin is shown below each gel lane. The ratios were normalized to control siRNA-transfected cells. (**C**) Cell viability was determined at 48 h post-transfection of distinct siRNA targeting CTSB, D or L by MTT assay. The siRNAs used were CTSB1 siRNA, CTSD2 siRNA, and CTSL2 siRNA. Results were averaged and blots were representative of 3 independent experiments. *P < 0.05, **P < 0.01, ***P < 0.001 as compared to control siRNA transfected-cells.

Given that obatoclax was trapped in lysosomes and caused their redistribution, this raised the possibility that obatoclax could also affect lysosomal functions. Cysteine proteases cathepsin B (CTSB) and L (CTSL), together with the aspartic protease cathepsin D (CTSD), are the most abundant lysosomal proteases [31]. To investigate the effect of obatoclax on lysosomal function, the active forms of these cathepsins were determined. Exposure for 48 h to obatoclax substantially lowered the levels of active CTSB, CTSD, and CTSL in HKESC-1 and HKESC-1/cis cells. In EC109 and EC109/CDDP cells wherein CTSB protein was undetectable, obatoclax similarly decreased the active forms of CTSD and CTSL (Figure 7A). Similar to the action of obatoclax, lysosomal inhibitors CQ or bafilomycin A1 reduced the protein levels of these cathepsins (Figure S5). To explore whether there was a connection between cathepsin expression and LC3-II accumulation, specific siRNAs targeting CTSB, CTSD and CTSL were utilized. Knockdown efficacy was verified by Western blots. Downregulation of CTSB, CSTD or CSTL substantially caused LC3-II accumulation (Figure 7B). Importantly, knockdown of CTSD or CTSL significantly suppressed cell viability in EC109 and EC109/CDDP cells. We did not test the effect of CTSB knockdown on cell viability because of the absence of CTSB in these cell lines. In HKESC-1 and HKESC-1/cis cells, knockdown of CTSB or CTSD significantly induced the loss of cell viability, whereas CTSL downregulation exerted no effect (Figure 7C). Thus, reduced levels of active cathepsins are likely to account for the loss of cell viability induced by obatoclax.

### Obatoclax synergized with proteasome inhibition

**Figure 8:**
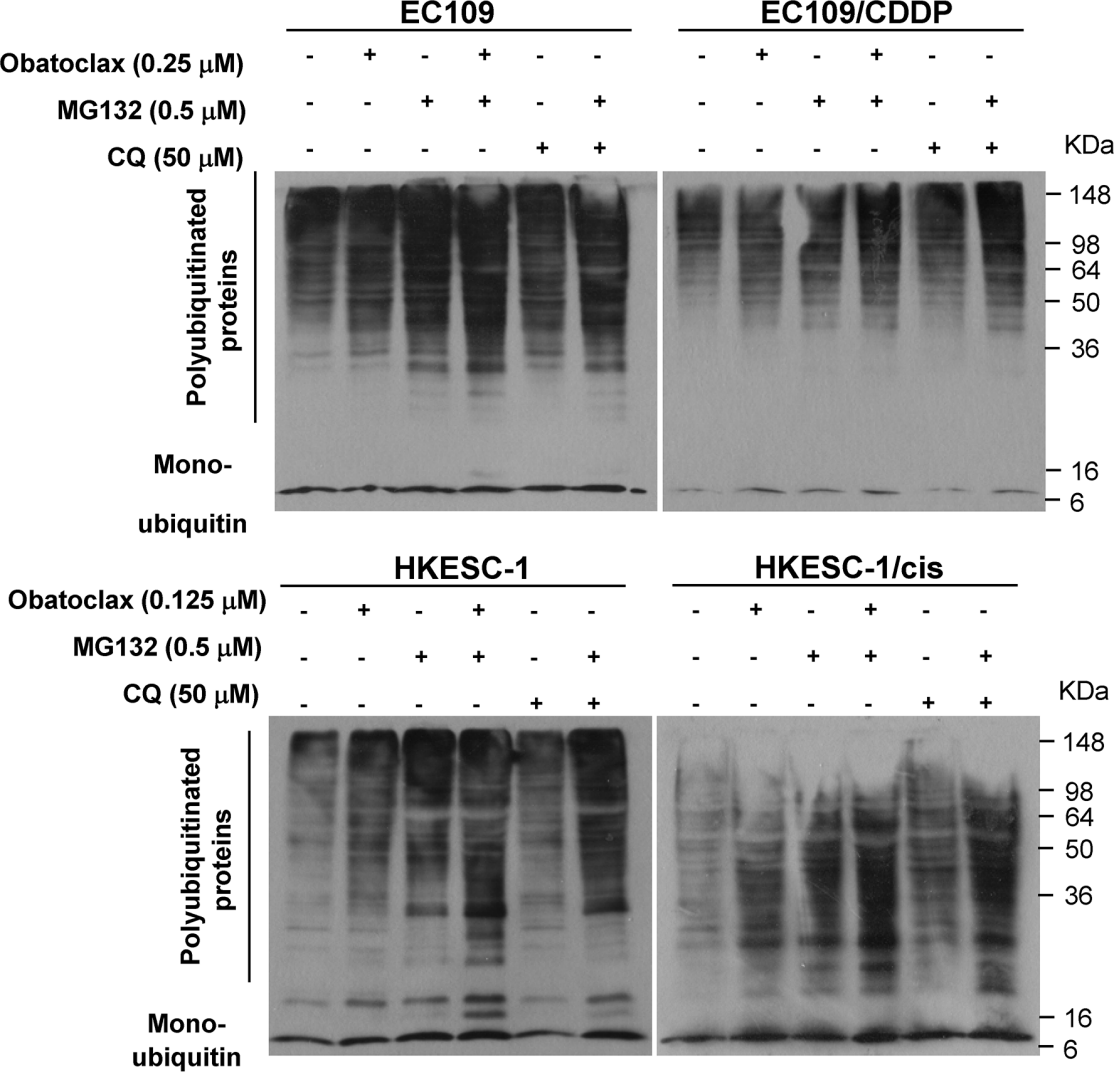
Accumulation of polyubiquitinated proteins caused by obatoclax in the absence or presence of MG132 Cells were treated with or without obatoclax or CQ at the indicated concentration in the absence or presence of MG132 for 48 h, and the amount of polyunbiquitinated proteins was examined by Western blot analysis. β-actin was used to evaluate protein loading. Blots were representative of 3 independent experiments.

Autophagy and the ubiquitin-proteasome system (UPS) are two complementary pathways for protein degradation. In this regard, the former mainly maintains cellular homeostasis by degradation of long-lived proteins and damaged organelles, but also helps to sequester and degrade polyubiquitinated protein aggregates [32, 33]. Consistent with the known function of autophagy and the effect of CQ, exposure for 48 h to obatoclax substantially induced the accumulation of polyubiquitinated proteins. Moreover, the combination of obatoclax or CQ with proteasome inhibitor MG-132 showed synergy in the accumulation of polyubiquitinated proteins (Figure 8), implying that the accumulation of polyubiquitinated proteins induced by obatoclax was caused by the block of autophagic degradation instead of inhibition of UPS.

## DISCUSSION

Although autophagy has been implicated in obatoclax-mediated cytotoxicity, the key question is whether this agent promotes or inhibits autophagic activity [21–28]. Also, the key biochemical events that link autophagy to the loss of cell viability upon obatoclax treatment have so far remained elusive. Here, we identified direct impairment of lysosomal function by obatoclax as a key event that connects the blockade of autophagy flux to the loss of cell viability, unveiling a novel mechanism of obatoclax-induced cytotoxicity. Because this action is presumed to bypass the mitochondrial pathway of apoptosis, obatoclax exhibits potent cytotoxicity in esophageal cancer cells regardless of their resistance to apoptosis.

In this study, we utilized cisplatin-resistant human esophageal cancer cell lines EC109/CDDP and HKESC-1/cis, which were obtained by prolonged culture of parental cell lines with sublethal cisplatin concentrations as previously described [34, 35]. Both cisplatin-resistant sublines manifested apoptosis resistance upon cisplatin challenge compared with their parental cell lines [35, 36]. Dysregulation of Bcl-2 family members have been associated with multiple instances of cisplatin resistance [13, 37, 38]. However, we did not observe a consistent upregulation or downregulation of Bcl-2 proteins in cisplatin-resistant cell lines, which might due to the limited number of cell lines used in our study. A recent study reported that cisplatin resistant lung cancer cells preserved sensitivity to apoptosis induction by obatoclax. This was likely to partially account for the equal effectiveness of obatoclax in cisplatin-sensitive and -resistant lung cancer cells [12]. However, our data showed that obatoclax as a single agent equally reduced cell viability in both cisplatin-sensitive and -resistant esophageal cancer cells, which was independent of apoptosis. Such discrepancy may reflect the heterogeneity of mechanisms of drug resistance in different cell types. Above all, our data suggested that obatoclax could mediate its cytotoxicity via a mechanism distinct from apoptosis, prompting us to investigate the role of the autophagy.

Unlike its differential effects on apoptosis induction, obatoclax exhibited autophagy-inhibitory activity in all tested cisplatin-sensitive and -resistant cancer cells, as shown by the increased levels of LC3-II and p62 and elevated numbers of LC3-positive puncta. The addition of CQ did not further elevate obatoclax-induced LC3- II protein expression in EC109 and EC109/CDDP cells, suggesting that increased LC3-II protein was caused by the blockade in autophagosome-lysosome fusion and/or lysosomal degradation rather than increased autophagosome formation. Similar to our observation, it has been reported that obatoclax combined with ERBB1/2 inhibitor lapatinib impaired autophagic degradation, reflected by accumulation of undigested large autophagosomes and LC3-II and p62 proteins [39].

Strikingly, widespread colocalization of obatoclax and lysosomes was observed, strongly implying that obatoclax was trapped in these organelles. The accumulation of obatoclax in lysosomes was concomitant with the redistribution of these organelles from a dispersed pattern to a clustered one. In agreement with our observation, the accumulation of lysosomes upon obatoclax treatment was also detected in breast cancer cells [25], suggesting the effect of obatoclax on lysosomes distribution may not be restricted to our tested cell lines. The mammalian homotypic fusion and vacuole protein sorting (HOPS) complex has been reported to participate in manipulating the clustering of lysosomes [40–42]. Whether HOPS is involved in obatoclax-induced clustering of lysosomes awaits further exploration. Obatoclax also caused swelling of mitochondria with loss of cristae structure. In line with this data, several Bcl-2 inhibitors including obatoclax have been reported to perturb mitochondria, suggesting these compounds might induce cell death by causing mitochondrial damage instead of specifically inhibiting Bcl-2 proteins [18]. However, whether mitochondrial swelling is reversible or contributes to obatoclax-induced cytotoxicity requires further study.

Consistent with our data, a recent study reported that obatoclax suppressed the expression of lysosomal hydrolases CTSD and CTSL [25]. Nevertheless the underlying mechanism is largely unknown. Cathepsins are synthesized as inactive precursors, which undergo proteolytic processing to produce the active, mature enzyme in the acidic environment of the lysosomes [31]. In the present study, obatoclax pronouncedly reduced the expression of mature CTSB, CTSD and CTSL, phenocopying the effect of lysosomotropic reagents CQ and bafilomycin A1, both of which impair the acidification of lysosomes [30]. Given that obatoclax is an indole-containing base, it is reasonable to postulate that it may function like CQ, when being trapped upon protonation in acidic compartments like lysosomes, it alkalinizes these compartments and thus compromises the proteolytic processing of cathepsins into their active forms. Loss of cell viability induced by cathepsin knockdown further connects the impaired lysosome function with the cytotoxicity of obatoclax. In this respect, both EC109 and EC109/CDDP cells were vulnerable to downregulation of CTSD or CTSL, whereas HKESC-1 and HKESC-1/cis cells were sensitive to CTSB or CTSD knockdown. These data suggest that different cell lines may rely on different cathepsins to maintain their normal autophagic flux, which is crucial to cell viability.

Autophagy takes part in the sequestration and degradation of polyubiquitinated protein aggregates [32, 33]. Pharmacological inhibition of autophagy by CQ, which alone increased the levels of polyubiquitinated proteins, could synergistically induce accumulation of polyubiquitinated proteins when combined with proteasome inhibitor MG132 [43]. Concordant with this report, we observed a similar phenomenon. Specifically, obatoclax phenocopied the effect of CQ on the accumulation of polyubiquitinated proteins.

In summary, our studies show for the first time that obatoclax is trapped in lysosomes. Its intralysosomal accumulation leads to the impairment of both lysosomal function and autophagic degradation capacity, inducing the loss of cell viability. Since this action is not reliant on the mitochondrial pathway of apoptosis, obatoclax substantially reduces cell viability in esophageal cancer cells irrespective of their susceptibility to cisplatin-induced apoptosis. Given that lysosomes have diverse cellular functions [42, 44, 45], whether obatoclax also affects other cellular events in addition to autophagic pathway awaits further exploration. Notably, the peak plasma concentration of obatoclax ranged from 14 to 155 ng/ml (34 to 375 nM) following 3-hour perfusion in clinical trials [46, 47], suggesting that the concentrations (0.125 and 0.25 μM) used in the present study are clinically achievable. The current findings not only provide novel insights into the molecular mechanism of obatoclax-induced cytotoxicity, but may also offer a rationale for the evaluation of obatoclax for the treatment of esophageal cancer patients, especially in combination with proteasome inhibitor and for those with cisplatin-resistant tumors.

## MATERIALS AND METHODS

### Reagents

Obatoclax was purchased from Selleck Chemicals (Houston, TX). Chloroquine, MG132 and bafilomycin A1 were obtained from Sigma-Aldrich (St. Louis, MO).

### Cell culture and cell viability assay

The HKESC-1 human esophageal cancer cell line and its cisplatin-resistant derivative HKESC-1/cis have been described in our previous study [35]. The EC109 and its cisplatin-resistant subline EC109/CDDP, another pair of human esophageal cancer cell lines utilized in this study, have been described previously [34, 36]. HKESC-1 and EC109 (parental and cisplatin-resistant) cells were maintained in MEM (Coring Cellgro, Manassas, MA) and RPMI1640 (Invitrogen, Carlsbad, CA) medium respectively, supplemented with 10% fetal bovine serum (Invitrogen), 100 U/mL penicillin and 100 μl/mL streptomycin (Invitrogen) at 37°C in a humidified atmosphere of 5% CO_2_ and 95% air. Cell viability was measured by MTT assay.

### Colony formation assay

Colony formation assay determines whether cells can recover from treatment and form colonies. After treatment, viable cells were re-seeded at the concentration of 3,000 cells per well in fresh media in six-well plates (in triplicate) and allowed to grow for 10–12 days. To visualize colonies, cells were fixed in absolute ethanol and stained with Hematoxylin Solution (Sigma-Aldrich). Colonies were then counted using ImageJ software.

### Annexin V labeling

After drug treatment, adherent cells were detached from culture dishes by treating with trypsin for 3–5 min and combined with floating cells. Apoptosis was measured using the FITC Annexin V Apoptosis Detection Kit (BD Biosciences, CA). The extent of apoptosis was quantified as percentage of Annexin V positive cells.

### Western blot analysis

After treatment, cells were washed with PBS and lysed with RIPA buffer with protease inhibitors (Merck, New York) and phosphatase inhibitors (Merck). Equal amounts of protein (40 μg/lane) were resolved by SDS-PAGE, and transferred to PVDF membrane (Roche, Indianapolis, IN). All primary antibodies were incubated overnight at 4°C: anti-Bcl-2 (2870), anti-Bcl-xL (2764), anti-Mcl-1 (5453), anti-Bad (9239), anti-Bid (2002), anti-Bim (2933), anti-Puma (12450), anti-Bak (12105), anti-Bax (5023), anti-ATG5 (8540), anti-ATG7 (2631), anti-Beclin-1 (3738), anti-β-actin (4970), anti-cathepsin D (2284), anti-LC3B (2775), anti-p62 (8025), anti-ubiquitin (3936) (Cell signaling, Beverly, MA), anti-cathepsin B (C6243) (Sigma-Aldrich), and anti-cathepsin L (BMS1032) (eBioscience, San Diego, CA). All primary antibodies were used at a dilution of 1:1000, except for anti-ATG7 protein, which was used at a dilution of 1:500. The membranes were then incubated for 1 h with secondary peroxidase-conjugated antibodies (1:2000) (Anti-rabbit antibodies, Cell Signaling Technology, 7074; Anti-mouse antibodies, Cell Signaling Technology, 7076). Chemiluminescent signals were then developed with Lumiglo reagent (Cell Signaling Technology, 7003) and exposed to X-ray film (FUJIFILM Europe GmbH, Dusseldorf, Germany). The films were analyzed by densitometry with ImageJ software.

### Immunofluorescence

Cells grown on petri-dish were fixed with 4% (v/v) paraformaldehyde for 30 min and then permeabilized with methanol at −20°C for 10 min. The cells were covered with 10% (v/v) goat serum (Invitrogen) for 30 min at room temperature to block nonspecific adsorption of antibodies to the cells. After blocking, cells were incubated with primary antibodies against LC3B or LAMP1 (Cell Signaling Technology, 9091) at a dilution of 1:400 at 4°C overnight. Cells were then probed with Alexa Fluoro 488 goat anti-rabbit secondary antibodies (1:500) (Invitrogen, A-24922) and incubated at room temperature for another 2 h. Fluorescent signals were determined using a Nikon C1-si confocal microscope, and images were analyzed with ImageJ software as described previously [48].

### Lysosomes labeling in live cells

After treatment, intracellular lysosomes were labeled and tracked using the CytoPainter LysoGreen Indicator Reagent (Abcam, Cambridge, MA) according to the manufacturer's protocol.

### RNA interference

The expression of ATG5 and ATG7 was lowered using predesigned target-specific siRNA oligonucleotides (ATG5-Hs_ATG5L_6, SI02655310, Hs_ATG5L_2, SI00069251; ATG7-Hs_ATG7_5, SI04952339, Hs_ATG7L_5, SI02655373) purchased from Qiagen (Hilden, Germany). The expression of Beclin-1, CTSB, D and L was downregulated by predesigned target-specific siRNA oligonucleotides (Beclin-1-SASI_Hs02_00336256, SASI_Hs01_00090914; CTSB-SASI_Hs01_00108033, SASI_Hs01_00108034, SASI_Hs01_00108035; CTSD- SASI_Hs02_00332783, SASI_Hs01_00141231, SASI_Hs01_00141234; CTSL- SASI_Hs01_00079399, SASI_Hs01_00079400, SASI_Hs02_00332791) from Sigma-Aldrich. Cells were seeded in 6-well plates. At 40–60% confluence, 150 ng of gene-specific siRNA or control siRNA (SI03650318, Qiagen) was transfected into cells using 12 μL HiperFect Transfection Reagent (Qiagen) according to the manufacturer's instruction. Nontargeting siRNA was used as control siRNA, which has no homology to any known mammalian gene.

### Electron microscopy

After treatment, cells were collected and fixed in 2.5% glutaraldehyde in 0.1 M phosphate buffer for 3 h at 4°C, followed by post-fixation in 1% osmium tetroxide for 1 h at room temperature. Samples were dehydrated in graded ethanol solutions, and infiltrated and embedded in Spurr's low-viscosity medium (TED PELLA, Redding, CA). Ultrathin sections of 60 nm were cut in a Leica microtome, double-stained with uranyl acetate and lead acetate, and examined in a Hitachi 7500 transmission electron microscopy at an accelerating voltage of 60 kV. Digital images were obtained using SIS Megaview III system.

### Statistical analysis

Results were expressed as mean ± S.E.M. of multiple experiments. Statistical analysis was performed with either an analysis of variance (ANOVA) followed by the Tukey's *t* test or Student's *t* test. *P* values less than 0.05 were considered statistically significant.

## SUPPLEMENTARY MATERIALS FIGURES


