# Supplementary material for: Obatoclax impairs lysosomal function to block autophagy in cisplatin-sensitive and -resistant esophageal cancer cells
Source: Oncotarget. 2016 Feb 19;7(12):14693–707. doi: 10.18632/oncotarget.7492 (PMC4924745; doi:10.18632/oncotarget.7492)
Supplement: Supplementary file 1 [file oncotarget-07-14693-s001.pdf]

Obatoclax impairs lysosomal function to block autophagy in cisplatin-sensitive and -resistant esophageal cancer cells

Supplementary Materials

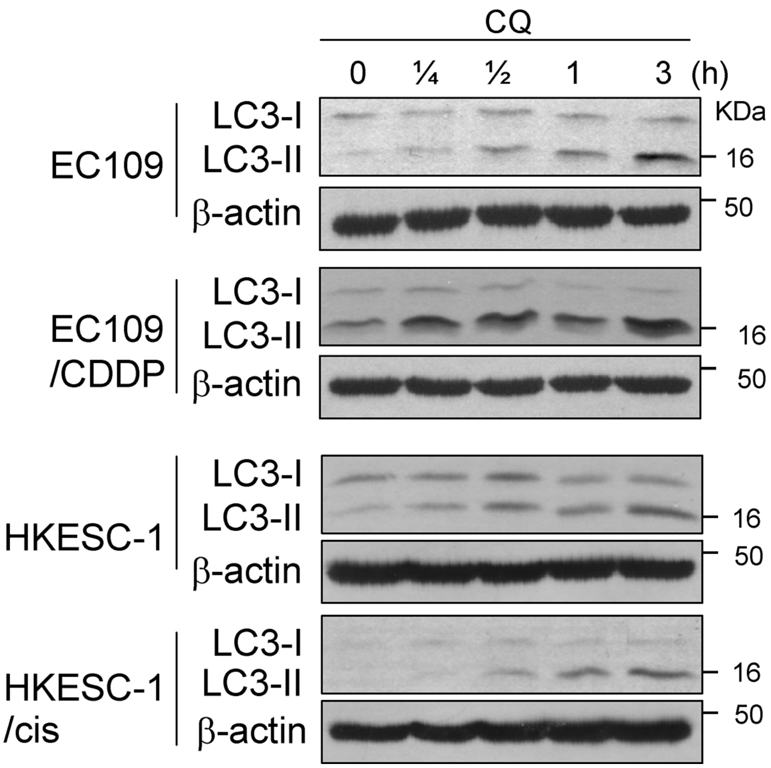

Supplementary Figure S1 (Related to Figure 3C): Chloroquine increased LC3-II expression. Cells were treated with 50 µM chloroquine (CQ) for the indicated time periods. The conversion of LC3-I to LC3-II was determined by Western blot analysis. Blots were representative of 3 independent experiments.

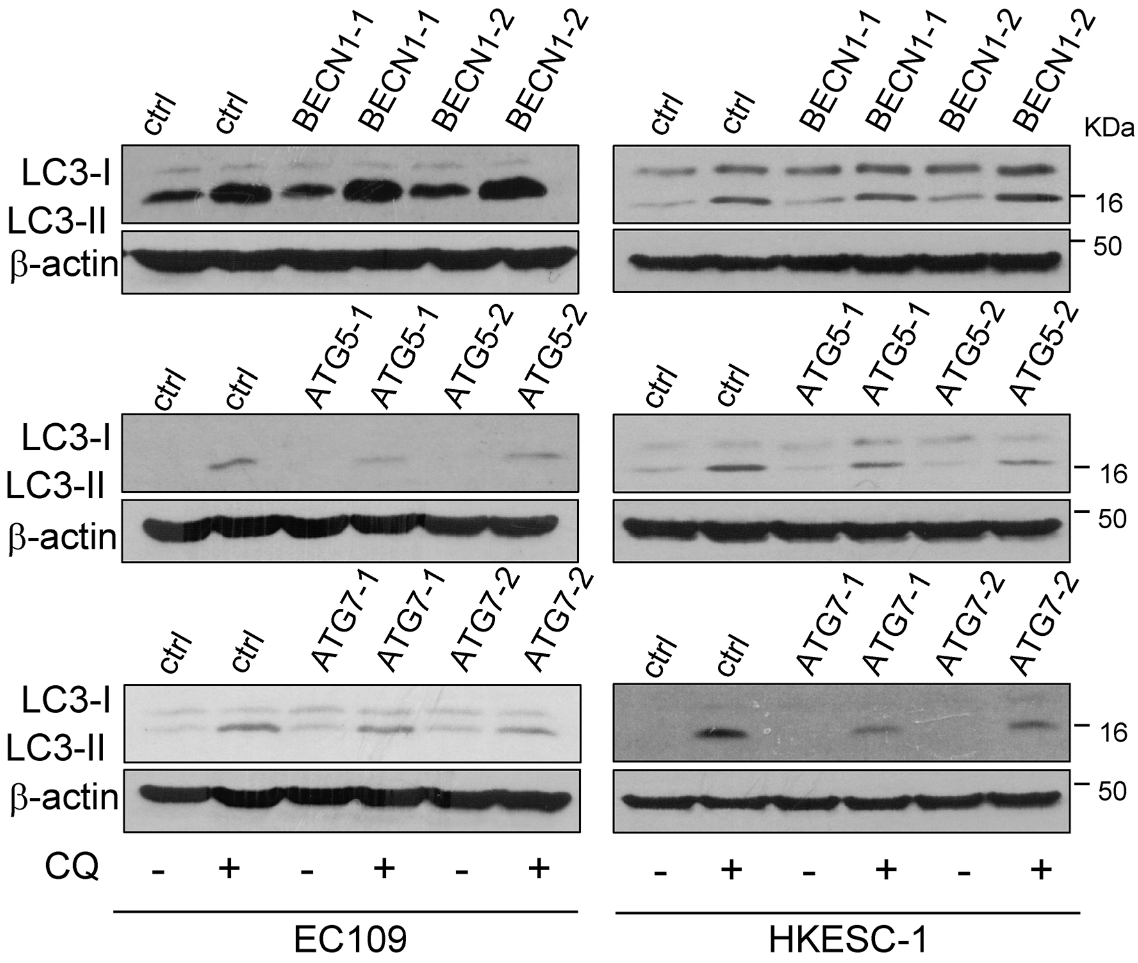

Supplementary Figure S2 (Related to Figure 5B): Effects of siRNA-mediated knockdown of Beclin-1, ATG5, or ATG7 on choloroqine-induced LC3-II accumulation. After transfection with the control siRNA, Beclin-1 siRNA, ATG5 siRNA, or ATG7 siRNA, cells were treated with obatoclax at the concentration of 0.25 μM (EC109 cells) and 0.125 μM (HKESC-1 cells) for 3 h. The conversion of LC3-I to LC3-II was determined by Western blot analysis. β-actin was used to evaluate protein loading. Blots were representative of 3 independent experiments.

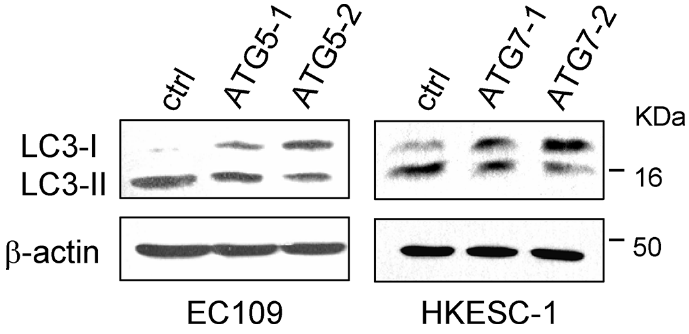

Supplementary Figure S3 (Related to Figure 5B): Downregulation of ATG5 or ATG7 decreased basal LC3-II expression. After transfection with the control siRNA, ATG5 siRNA or ATG7 siRNA, cells were collected. The conversion of LC3-I to LC3-II was determined by Western blot analysis. β-actin was used to evaluate protein loading. Blots were representative of 3 independent experiments.

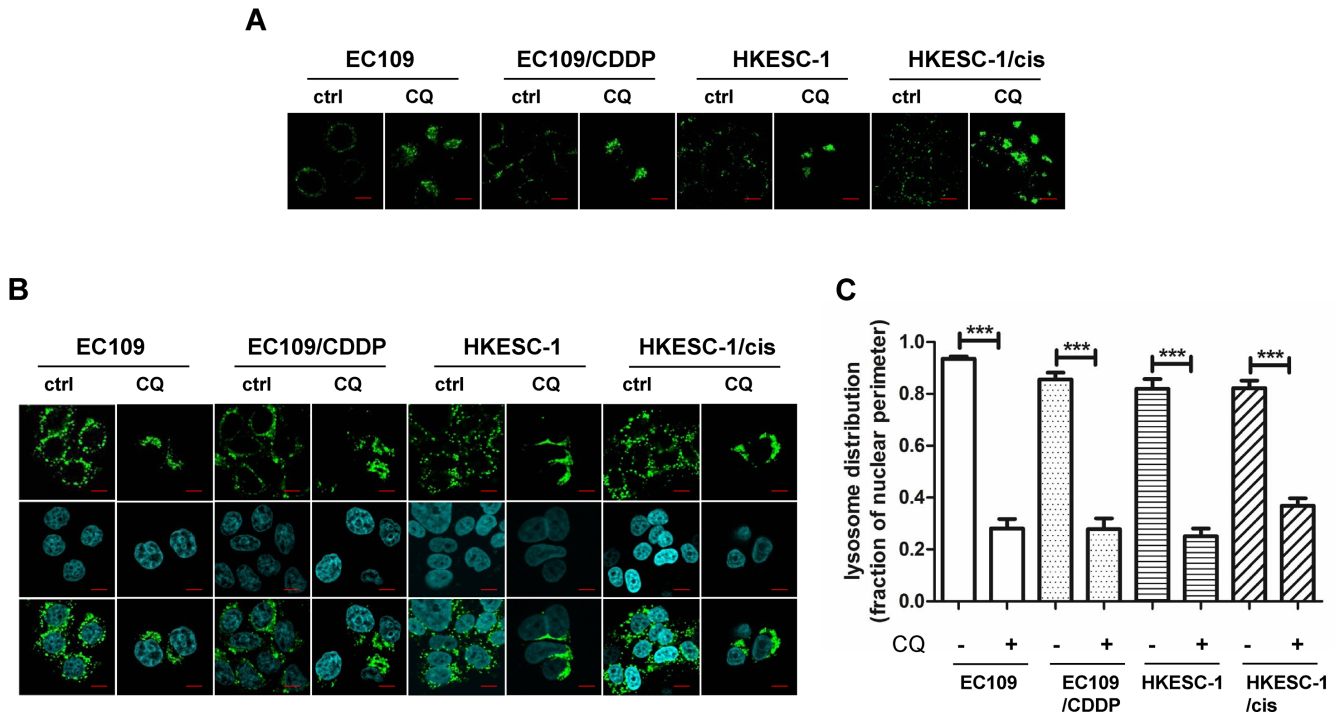

Supplementary Figure S4 (Related to Figure 6): Chloroquine induced lysosome clustering. (**A**) Cells were treated with 50 μM chloroquine (CQ) for 3 h. Lysosomes in live cells were stained with LysoGreen Indicator reagent. Scale bars, 10 μm. (**B**) Cells were treated with 50 μM CQ for 3 h. Immunofluorescent staining for LAMP1 marked lysosomes. The results shown are representative images. Scale bars, 10 μm. (**C**) Quantification of lysosome distribution (lysosome distribution relative to nuclear perimeter) is shown. For each point, at least 30 cells were pooled from three independent experiments. ****P* < 0.001 as compared with control cells.

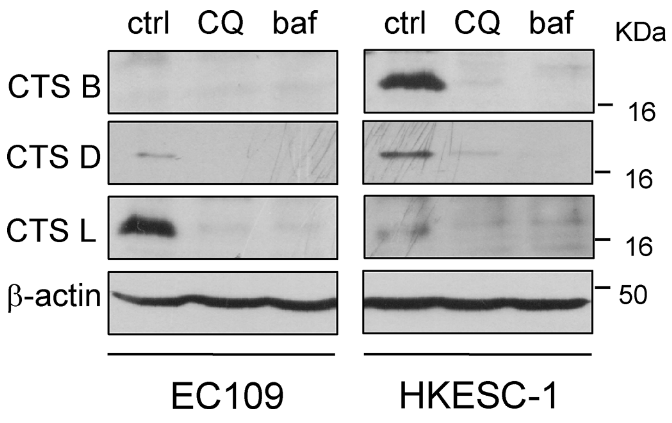

Supplementary Figure S5 (Related to Figure 7A): Effects of lysosomatropic agents chloroquine and bafilomycin A1 on protein levels of cathepsin B (CTSB), cathepsin D (CTSD), or cathepsin L (CTSL). Cells were treated with chloroquine (50 μM) or bafilomycin A1 (10 nM) for 48 h. The mature CTSB, D and L expression was determined by Western blot analysis. Blots were representative of 3 independent experiments.
